# A rare case of middle cerebral artery aneurysm

**DOI:** 10.11604/pamj.2021.39.241.30870

**Published:** 2021-08-15

**Authors:** Medhavi Vivek Joshi, Rashmi Ramesh Walke

**Affiliations:** 1 Ravi Nair Physiotherapy College, Datta Meghe Institute of Medical Sciences, Sawangi, Wardha, Maharashtra, India

**Keywords:** Aneurysm coiling, digital subtraction angiography, embolization

## Image in medicine

A 40-year-old female, presented with complaints of severe headache since past five days. She is a known case of controlled hypertension. Multislice computed tomography (CT) angiography-brain arteries was done which revealed abnormal high attenuation lesions of blood Hounsfield units (HU), seen in the basal cisterns, suprasellar cisterns, right sylvian fissure and few cortical sulci of right cerebral hemisphere were suggestive of subarachnoid haemorrhage. It also revealed right Middle cerebral artery aneurysm measuring 1.13 x 1.02cm. Surgical management through aneurysm coiling and embolization was done.

**Figure 1:**
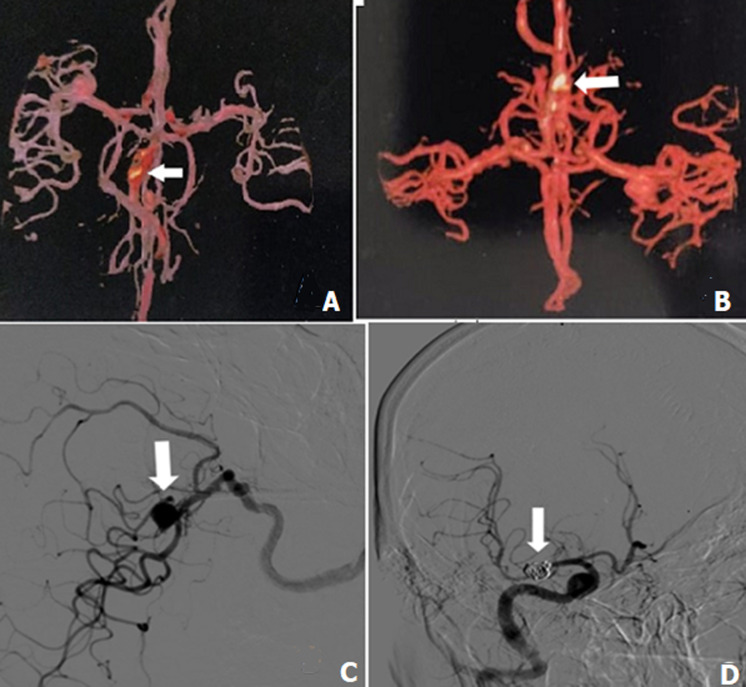
showing angiography reports pre and post aneurysm coiling and embolization; A,B) pre-operative multi-slice CT angiography of Brain arteries revealing Aneurysm in right middle cerebral artery; C) digital subtraction angiography (intravenous) pre-operative, showing right middle cerebral artery aneurysm (arrows); D) post-operative digital intravenous angiogram showing (arrow) coiling of the aneurysm and embolization visible through restricted blood flow to the coiled area

